# Supplementary material for: Patterns of Intron Gain and Loss in Fungi
Source: PLoS Biol. 2004 Nov 30;2(12):e422. doi: 10.1371/journal.pbio.0020422 (PMC532390; doi:10.1371/journal.pbio.0020422)
Supplement: Table S1 — Also available at http://genes.mit.edu/NielsenEtAl/. (4.3 MB ZIP). [file pbio.0020422.st001.zip › NielsenEtAl/html/883.html]

AN5793.1.NCU02493.1.MG07727.1.FG00564.1


```
 CLUSTAL W (1.82) Multiple Sequence Alignments - Introns Inserted


Sequence 1: NCU02493.1	297 aa
Sequence 2: MG07727.1	292 aa
Sequence 3: FG00564.1	296 aa
Sequence 4: AN5793.1	298 aa
Alignment Length: 301 aa
Number Identitical Residues: 186 aa
Alignment Score (without introns) 8419


MG07727.1 	M0TSIGTGYDLANSIFSPDGRNFQ~VEYAVKAVENGGTSVGIRCKDGVVLAVEKVVTSKL
NCU02493.1	M0TSIGTGYDLANSIFSPDGRNFQ~VEYAMKAVENGSTSIGIRCKDGIVLAAEKVITSKL
FG00564.1 	M0TSIGTGYDLLNSIFSPDGRNFQ~VEYAVKAVESGGTSIGIRAKDGVVLAIEKVVSSKL
AN5793.1  	M0TSIGTGYDLSNSVFSPDGRNFQ0VEYAVKAVENGGTSIGIRCKDGVVLAVEKIITSKL
          	* ********* **:********* ****:****.*.**:***.***:*** **:::***

MG07727.1 	LKPGANKRIATVDRHLGV0AYSGMIPDGRHFVDRARDEARSWRDTFKTPIGTPELASRMG
NCU02493.1	LKQGANKRIATVDRHLGI0VYSGMIPDGRHFVDRARDEARGWRDNYKTPISTKDLAGRMG
FG00564.1 	LKPGANKRIATVDSHVGA0VSSGMVPDGRHFVDRARDEAQSWRQNFKTPIPTSDLASRMG
AN5793.1  	LKPGANKRISTVDRHVGI0VSSGLAPDGRHFVSRARDEASSWRGIYKSPIPVSALANRLG
          	** ******:*** *:*  . **: *******.****** .**  :*:** .  **.*:*

MG07727.1 	GYLQAYTLYQSVRPFGITAIVAGWDSSLELPVDAEVGVGPSCGAGGKSTDKKYGGPQLYM
NCU02493.1	GYMQAYTLYQSVRPFGITAIIGGYDSELDTPVEGEVGSGPSVGAGGKVEGKKHGGPFLYM
FG00564.1 	GYLQAYTMYGSVRPFGITAILGGYDTPEETPVDGEVGSGPKVGAGGKVEG-KHGGPFLYM
AN5793.1  	GYVQAYTLYSSVRPFGVTSIVGGWDSEAELAVDGQVGSGPQSGSGGKVEGAKSGGPGLYM
          	**:****:* ******:*:*:.*:*:  : .*:.:** **. *:***  . * *** ***

MG07727.1 	IEPSGLYW~GYYGAATGKGRQAAKAELEKLDLSGDG---ISLEQAVKEAARIIYVAHDDN
NCU02493.1	VEPSGLYW~GYYGAATGKGRQAAKAELEKLDLSSDGESSLTLEQAVKEAARIIYVAHDDN
FG00564.1 	IEPSGMYW~GYYGAATGKGRQVAKAELEKLDLAAGN---MSLEDAVKQAARIIYIAQKDN
AN5793.1  	IEPSGLYW0GYYGAATGKGRQAAKAELEKLDLASEK---LSLQDAVKEAARIIYVAHEDN
          	:****:** ************.**********:.     ::*::***:******:*:.**

MG07727.1 	KDKEFELEMTWISSEDGPTKGRHEEVPKKLLEEAERLAKKAIEGDDDDDDD-KDD-----
NCU02493.1	KDKEFELEMTWISGADGPTKGRHQEVPEALRKEAEKYAERMTAKDIEEEEE-TKEGG---
FG00564.1 	KDKDFELEMTWISGPDGPTKGRHVEVPKELREEAERLAKAEDEDDDDDDDD-DNEDAKDD
AN5793.1  	KDKEFELEMSWISSLNGPTKGRHEEVPKELLEEAERAAKRALEGDDEEEEDGSKATGNEG
          	***:*****:***. :******* ***: * :***: *:     * :::::. .  ....

MG07727.1 	EKMEE
NCU02493.1	DKMEE
FG00564.1 	DKMED
AN5793.1  	ERMEE
          	::**:
```
